# Anthropometric predictors of hypertension among Moroccan adults: a cross-sectional study

**DOI:** 10.3389/fpubh.2026.1781362

**Published:** 2026-04-10

**Authors:** Chourite Chaimaa, Khossossi Hanae, Eljakani Elhoucine, Fedouache Mohammed, Challal Ismail, Zaguiri Mohammed, Mouilly Mustapha

**Affiliations:** 1 Interdisciplinary Laboratory of Sport Sciences, Institute of Sport Profession, Ibn Tofail University, Kenitra, Morocco; 2 Minister of Health and Social Protection, Fes, Morocco; 3 Faculty of Sciences, Ibn Tofail University, Kenitra, Morocco; 4 Higher Institute of Nursing Professions and Health Techniques, Casablanca, Morocco

**Keywords:** anthropometric indices, cardiovascular risk, hypertension, Morocco, WHtR

## Abstract

**Background:**

Hypertension remains a major public health challenge worldwide, particularly in low- and middle-income countries. Obesity-related anthropometric indicators are widely used to assess cardiovascular risk; however, evidence regarding the most reliable predictors of hypertension remains inconsistent across populations. Therefore, this study aimed to determine the prevalence of overweight and obesity based on general and central adiposity indicators, assess the association between multiple anthropometric measures and hypertension, and identify the most reliable predictor of hypertension among Moroccan adults.

**Methods:**

400 Moroccan adults were involved in this cross-sectional study. Sociodemographic, lifestyle, clinical, and anthropometric data were collected. Hypertension was defined according to international guidelines. Receiver operating characteristic (ROC) curve analysis was used to assess discriminatory performance, with optimal cut-off values determined using the Youden index. Multivariable logistic regression models were applied to identify independent associations.

**Results:**

Hypertension was present in 75.0% of participants. The prevalence of overweight and obesity was 58.3 and 8.8%, respectively, indicating that 67.1% of the study population had excess body weight. Waist-to-height ratio (WHtR) showed the strongest independent association with hypertension (ORa = 6.79, 95% CI: 3.57–12.90, *p* < 0.001). In ROC analysis, WHtR demonstrated the highest discriminatory performance (AUC = 0.793, 95% CI: 0.744–0.842), outperforming waist-to-hip ratio (AUC = 0.657,95% CI: 0.596–0.718), body fat percentage (AUC = 0.603, 95% CI: 0.538–0.668), visceral fat level (AUC = 0.584, 95% CI: 0.525–0.642), and body mass index (AUC = 0.568, 95% CI: 0.503–0.633). The optimal WHtR cut-off value of 0.505 yielded a sensitivity of 79.7% and specificity of 63.0%.

**Conclusion:**

Waist-to-height ratio, appears to be the most reliable anthropometric predictors of hypertension among Moroccan adults. Given its simplicity and low cost, WHtR may represent a practical tool for community-based screening and early identification of individuals at increased risk of hypertension in Morocco.

## 1 Introduction

Hypertension is a leading cause of morbidity and mortality worldwide, accounting for over 10 million deaths annually, particularly in low- and middle-income countries (1, 2). In Morocco, recent epidemiological data suggest that the prevalence of hypertension exceeds 30% among adults, with a significant proportion of cases remaining undiagnosed or inadequately controlled (3). Despite advances in antihypertensive pharmacotherapy, a substantial proportion of patients continue to experience uncontrolled blood pressure, highlighting persistent therapeutic gaps and the need for improved early identification strategies (4).

Obesity is one of the most significant modifiable risk factors for hypertension, and its assessment through anthropometric indicators has become central to clinical and public health practices (5, 6). Conventional measures such as body mass index (BMI), waist circumference (WC), waist-to-hip ratio (WHR), and waist-to-height ratio (WHtR) are widely used as proxies for body composition and adiposity (7, 8). While BMI is the most frequently applied indicator, it does not differentiate between fat and lean mass or account for fat distribution, often underestimating cardiovascular risk in individuals with normal weight but excess visceral fat (9, 10). In contrast, central obesity measures WC, WHR, and WHtR are more strongly associated with adverse cardiometabolic outcomes, including hypertension (11). These indices reflect abdominal fat accumulation, which is closely linked to insulin resistance, inflammation, sympathetic nervous system activation, and other mechanisms involved in the pathogenesis of elevated blood pressure (12).

From a pathophysiological perspective, excess adiposity contributes to hypertension through metabolic, inflammatory, and vascular mechanisms. Recent metabolomic studies have demonstrated disturbances in lipid and inflammatory pathways, including linoleic and arachidonic acid metabolism, in hypertensive individuals (13) Moreover, endothelial dysfunction and extracellular matrix remodeling mediated by matrix metalloproteinases contribute to vascular stiffness and increased peripheral resistance (14). Vascular dysfunction, including impaired vasodilation and salt-sensitive blood pressure responses, may be further exacerbated by dietary and lifestyle transitions (15). Although pharmacological and complementary approaches for blood pressure control continue to be investigated, their effects remain heterogeneous, underscoring the importance of prevention and early risk detection (16).

In Morocco, hypertension represents a major and growing public health concern, with recent estimates indicating that approximately 36.9% of adults are affected (17). This high prevalence is occurring within a context of rapid demographic and epidemiological transition (18), characterized by urbanization, population aging, and substantial shifts in lifestyle behavior (19, 20). hypertension represents a major and growing public health concern. Changes in dietary patterns, increased consumption of energy-dense foods, reduced physical activity, and rising sedentary behavior have profoundly influenced body composition and cardiometabolic risk profiles in the Moroccan population. These transformations have contributed to a parallel increase in both general and central obesity, further amplifying the burden of hypertension and related cardiovascular complications.

Despite these developments, limited research has investigated the relationship between anthropometric indicators and hypertension in the Moroccan context. Most available evidence originates from Western and Asian populations, limiting its generalizability to North African settings where cultural, genetic, and environmental factors may modulate these associations (21). Moreover, recent advances in body composition analysis such as bioelectrical impedance analysis which can provide more precise assessments of visceral fat and body fat percentage, are seldom used in epidemiological research in the region (22, 23).

Given the increasing burden of hypertension and obesity in Morocco, there is a critical need to identify reliable, simple, and context-appropriate anthropometric indicators for hypertension risk assessment. In this context, it is also important to quantify the prevalence of overweight and obesity using standardized general and central adiposity measures to better characterize population risk. Therefore, this study aims to determine the prevalence of overweight and obesity based on anthropometric indicators, examine the association between multiple anthropometric indices and hypertension, and identify the most reliable predictor of hypertension among Moroccan adults.

## 2 Materials and methods

### 2.1 Study design and population

This study was designed as an analytical cross-sectional. The reporting of this study followed the Strengthening the Reporting of Observational Studies in Epidemiology (STROBE) guidelines (24).

Participants were consecutively recruited during routine clinical consultations and anthropometric assessments conducted at a primary healthcare center in the province of Kenitra, Morocco, between July and November 2025. All adults attending the center during the study period were invited to participate. After recruitment, eligibility criteria were applied to determine inclusion in the study. Individuals who met the predefined inclusion criteria and did not present any exclusion criteria were enrolled in the final study sample. Following written informed consent, standardized anthropometric and blood pressure measurements were performed. Based on these measurements, participants were subsequently classified into normotensive and hypertensive groups according to international hypertension guidelines. Individuals with systolic blood pressure ≥140 mmHg and/or diastolic blood pressure ≥90 mmHg, or those reporting current use of antihypertensive medication, were classified as hypertensive, while participants with systolic blood pressure <140 mmHg and diastolic blood pressure <90 mmHg and not receiving antihypertensive treatment were classified as normotensive.

Inclusion criteria comprised adults aged ≥18 years who voluntarily agreed to participate, provided written informed consent, and completed standardized anthropometric and blood pressure assessments, with residency in the Kenitra province for at least 6 months.

Exclusion criteria included pregnancy; use of a pacemaker or other implanted electronic devices; physical or musculoskeletal conditions preventing accurate standing measurements; severe acute or chronic diseases such as advanced heart failure, renal failure, active malignancy, or acute infectious conditions; known endocrine disorders affecting body composition; Use of corticosteroid therapy; inability to provide reliable questionnaire responses; and incomplete key data, including missing blood pressure or anthropometric measurements.

The required sample size was calculated using the standard formula for cross-sectional studies [n = Z^2^pq/δ^2^], assuming a hypertension prevalence of 30%, a 95% confidence level, and a permissible error of 10% of the prevalence. The minimum sample size was estimated at 385 participants and was increased to 400 to account for potential missing data. a minimum of 385 participants was required. This number was intentionally increased to 400 to enhance statistical power, ensuring adequate precision of the estimates. According to a previous study the prevalence of hypertension in Morocco ranged from 26.6 to 33.6% (3, 25). The study was conducted according to the guidelines of the Helsinki Declaration. The protocol of this study was approved by the Ethics Committee of the Faculty of Medicine and Pharmacy, Mohammed V University of Rabat.

### 2.2 Measurements and definitions

#### 2.2.1 Dependent variable

Blood pressure was measured using a validated automated digital sphygmomanometer (Omron, Kyoto, Japan) with an appropriately sized cuff. Measurements were performed with participants seated comfortably, after at least 5 min of rest, with the back supported and feet flat on the floor. Two measurements were taken at 1–2 min intervals on the same arm, and the average of the two readings was used for analysis. Heart rate (beats per minute) was automatically recorded by the device at the time of blood pressure measurement. Status was defined according to European Society of Cardiology guidelines (26) as systolic blood pressure ≥ 140 mmHg and/or diastolic blood pressure ≥ 90 mmHg. Participants were classified as hypertensive or normotensive accordingly.

#### 2.2.2 Independent variables

Anthropometric measurements: Body weight and body composition parameters were assessed using a validated bioelectrical impedance analysis device (InBody HN20, InBody Co., Seoul, Korea) (27). according to the manufacturer’s standardized procedures. The variables obtained from the BIA device included body fat percentage (%), skeletal muscle percentage (%), and visceral fat level. Height was measured using a portable stadiometer, with participants standing barefoot, in an upright position. Waist circumference (WC) and hip circumference (HC) were measured using a non-elastic anthropometric measuring tape following World Health Organization (WHO) standardized protocols (28). WC was measured at the midpoint between the lowest rib and the iliac crest, while HC was measured at the level of the greatest posterior protuberance of the buttocks. From these measurements WHR, and WHtR were calculated.

#### 2.2.3 Covariates

Potential confounding variables included age, sex, educational level, income level, marital status, smoking status, alcohol consumption, physical activity level. These variables were selected based on prior epidemiological evidence. Sociodemographic and lifestyle data were collected using a structured, interviewer-administered questionnaire. Physical activity level was assessed using a validated standardized questionnaire adapted for the Moroccan population. Smoking status and alcohol consumption were categorized into predefined groups based on frequency and current use. Age and sex were recorded directly, and educational level, income level, and marital status were self-reported by participants.

### 2.3 Statistical analysis

Statistical analyses were performed using IBM SPSS Statistics version 26.0. The normality of continuous variables was assessed using the Shapiro–Wilk test. Normally distributed variables were expressed as mean ± standard deviation, whereas non-normally distributed variables were presented as median (interquartile range); categorical variables were summarized as frequencies and percentages. Group comparisons between hypertensive and normotensive participants were conducted using the independent samples t-test or Mann–Whitney U test, as appropriate, and the Chi-square test for categorical variables.

For logistic regression analyses, anthropometric indicators were categorized using established standard cut-off points. Binary logistic regression was performed in two stages: Model 1 estimated crude odds ratios between categorized anthropometric variables and hypertension, and Model 2 adjusted for potential confounders including age, sex, educational level, income level, marital status, smoking status, alcohol consumption, and physical activity level. Adjusted odds ratios with 95% confidence intervals were reported.

Model discrimination was evaluated using receiver operating characteristic (ROC) curve analysis. Optimal cut-off values were determined using the Youden index (J = sensitivity + specificity − 1), selecting the threshold that maximized the combined sensitivity and specificity for each anthropometric indicator. A *p*-value < 0.05 was considered statistically significant.

## 3 Results

### 3.1 Basic characteristics of study participants

In this cross-sectional sample of 400 adults, 300 participants with hypertension (75.0%) were significantly older than normotensive individuals (57.9 ± 7.7 vs. 44.9 ± 6.8 years, *p* < 0.001), while sex distribution did not differ between groups (*p* = 0.644). Hypertension was significantly associated with marital status, educational attainment, income level, and physical activity level (*p* < 0.001). No significant differences were observed for smoking status or alcohol consumption (Table 1).

**Table 1:** Sociodemographic, clinical and anthropometric characteristics of the participants. (*N* = 400).

Characteristics	Total (*n* = 400)	Normotensive (*n* = 100)	Hypertensive (*n* = 300)	*p*
Age, years	54.6 ± 9.4	44.9 ± 6.8	57.9 ± 7.7	<0.001
Gender, Female *(%)*	212 (53.0)	55 (55.0)	157 (52.3)	0.644
Marital status, *n* (%)				<0.001
Single	111 (27.8)	45 (45.0)	66 (22.0)	
Married	204 (51.0)	46 (46.0)	158 (52.7)	
Divorced	54 (13.5)	7 (7.0)	47 (15.7)	
Widowed	31 (7.8)	2 (2.0)	29 (9.7)	
Education level, *n* (%)				<0.001
No formal education	99 (24.8)	2 (2.0)	97 (32.3)	
Primary education	131 (32.8)	23 (23.0)	108 (36.0)	
Secondary education	101 (25.3)	41 (41.0)	60 (20.0)	
Higher education	69 (17.3)	34 (34.0)	35 (11.7)	
Income level, *n* (%)				<0.001
Low income	201 (50.2)	24 (24.0)	177 (59.0)	
Middle income	141 (35.3)	47 (47.0)	94 (31.3)	
High income	58 (14.5)	29 (29.0)	29 (9.7)	
Physical activity level, *n* (%)				<0.001
Low	185 (46.3)	24 (24.0)	161 (53.7)	
Moderate	158 (39.5)	56 (56.0)	102 (34.0)	
High	57 (14.2)	20 (20.0)	37 (12.3)	
Smoking status, *n* (%)				0.961
Never smoker	278 (69.5)	70 (70.0)	208 (69.3)	
Former smoker	79 (19.8)	20 (20.0)	59 (19.7)	
Current smoker	43 (10.8)	10 (10.0)	33 (11.0)	
Alcohol consumption, *n* (%)				0.635
None	198 (49.5)	49 (49.0)	149 (49.7)	
Rare	149 (37.3)	39 (39.0)	110 (36.7)	
Moderate	27 (6.8)	8 (8.0)	19 (6.3)	
Frequent	26 (6.5)	4 (4.0)	22 (7.3)	
BMI (kg/m^2^)				0.065
Normal weight	130 (32.5)	43 (43.0)	87 (29.0)	
Overweight	233 (58.3)	47 (47.0)	186 (62.0)	
Obesity	35 (8.8)	9 (9.0)	26 (8.7)	
WC (cm)	91.6 ± 7.4	88.5 ± 7.6	92.7 ± 7.0	<0.001
HC (cm)	102 (97.5–106.5)	102 (97.5–106.5)	102 (97.5–106.5)	0.694
WHtR	0.53 ± 0.04	0.49 ± 0.04	0.54 ± 0.04	<0.001
WHR	0.86 ± 0.05	0.83 ± 0.05	0.86 ± 0.05	<0.001
Body fat (%)	28.6 ± 5.1	27.1 ± 5.2	29.1 ± 5.0	0.001
Skeletal muscle (%)	36.2 ± 3.9	37.1 ± 3.5	35.9 ± 3.9	0.006
Visceral fat level	8 (7.0–9.0)	7 (6.0–9.0)	8.0 (6.0–9.0)	0.011
SBP (mmHg)	146.63 ± 17.79	122.26 ± 8.57	154.75 ± 11.54	<0.001
DBP (mmHg)	92.87 ± 11.26	78 ± 5.82	97.82 ± 7.71	<0.001
HR (beats/min)	73 ± 8	69 ± 7	75 ± 8	<0.021

n, number of participants. SBP, systolic blood pressure; DBP, diastolic blood pressure; HR, heart rate; BMI, body mass index; WHtR, waist-to-height ratio; WHR, waist-to-hip ratio.

Regarding lifestyle factors, physical activity levels differed significantly between groups (*p* < 0.001). Participants with hypertension were more frequently categorized as having low physical activity, whereas normotensive adults more commonly reported moderate or high activity levels. In contrast, smoking status and alcohol consumption did not differ significantly between the two groups (*p* > 0.05).

With respect to body mass index categories, the distribution showed a high prevalence of excess body weight in the study population. Overall, 58.3% of participants were classified as overweight and 8.8% as obese, indicating that 67.1% of adults had excess body weight. Overweight was more frequent among individuals with hypertension (62.0%) compared with normotensive participants (47.0%), whereas normal weight was more common among normotensive adults (43.0% vs. 29.0%). However, the overall distribution of BMI categories showed a limited association with hypertension status (*p* = 0.065).

In terms of anthropometric and body composition characteristics, individuals with hypertension exhibited significantly greater central and total adiposity compared with normotensive participants. Waist circumference was significantly higher among hypertensive individuals (92.7 ± 7.0 vs. 88.5 ± 7.6 cm, *p* < 0.001). Similarly, WHtR (0.54 ± 0.04 vs. 0.49 ± 0.04, p < 0.001) and WHR (0.86 ± 0.05 vs. 0.83 ± 0.05, *p* < 0.001) were significantly elevated in the hypertensive group. Body fat percentage was also higher among hypertensive participants (29.1 ± 5.0% vs. 27.1 ± 5.2%, *p* = 0.001), whereas skeletal muscle percentage was significantly lower (*p* < 0.05). Visceral fat levels were significantly higher among hypertensive individuals (median 8.0 vs. 7.0, *p* = 0.011).

Table 1 show that blood pressure measurements differed between groups. Participants with hypertension had significantly higher systolic blood pressure (154.75 ± 11.54 vs. 122.26 ± 8.57 mmHg, *p* < 0.001) and diastolic blood pressure (97.82 ± 7.71 vs. 78.00 ± 5.82 mmHg, *p* < 0.001) compared with normotensive participants. Resting heart rate was also higher among hypertensive individuals (75 ± 8 vs. 69 ± 7 beats/min, *p* = 0.021).

### 3.2 Association between anthropometric indicators and hypertension

Table 2 present the results of the logistic regression models adjusted for age, sex, income level smoking status and alcohol consumption, central adiposity indicators demonstrated the strongest and most consistent associations with hypertension. WHtR emerged as the most powerful predictor (ORa = 6.79; 95% CI: 3.57–12.90; *p* < 0.001), explaining a substantial proportion of variance (*R*^2^ = 0.693), followed by visceral fat (ORa = 5.95; 95% CI: 3.32–10.66; *p* < 0.001; *R*^2^ = 0.734) and WHR (ORa = 2.54; 95% CI: 1.64–3.94; *p* < 0.001; *R*^2^ = 0.641). In contrast, body fat percentage showed a modest but significant association with hypertension (ORa = 1.07; 95% CI: 1.01–1.15; *p* = 0.026), while BMI and skeletal muscle percentage were not independently associated with hypertension after adjustment (*p* > 0.05).

**Table 2:** Multivariable logistic regression model for the association between anthropometric measures and hypertension.

Variables	*R* ^2^	Adjusted OR	95% CI	*p*
BMI (kg/m^2^)	0.602	1.09	0.97–1.23	0.153
WHtR	0.693	6.79	3.57–12.90	<0.001
WHR	0.641	2.54	1.64–3.94	<0.001
Body fat (%)	0.609	1.07	1.01–1.15	0.026
Skeletal muscle (%)	0.604	0.93	0.85–1.01	0.097
Visceral fat level	0.734	5.95	3.32–10.66	<0.001

ORa, adjusted odds ratio; CI, confidence interval; WHtR, waist-to-height ratio; WHR, waist-to-hip ratio; BMI, body mass index. Model 1: unadjusted. Model 2: adjusted for age, sex, educational level, income level, marital status, smoking status, alcohol consumption, and physical activity level.

The correlation analyses showed significant positive correlations between blood pressure parameters and most anthropometric indicators. The strongest correlations with systolic blood pressure were observed for WHtR (*r* = 0.42, *p* < 0.001) and WHR (*r* = 0.40, *p* < 0.001), while WHtR also showed the strongest correlation with diastolic blood pressure (*r* = 0.37, *p* < 0.001) (Table 3).

**Table 3:** Correlation between anthropometric indicators and blood pressure parameters among participants (*N* = 400).

Variable	SBP (mmHg) r	*p*-value	DBP (mmHg) r	*p*-value
BMI (kg/m^2^)	0.18	0.02	0.16	0.05
WHtR	0.42	<0.001	0.37	<0.001
WHR	0.40	<0.001	0.21	0.002
Body fat (%)	0.20	0.001	0.17	0.004
Visceral fat level	0.26	<0.001	0.23	<0.001

SBP, systolic blood pressure; DBP, diastolic blood pressure; BMI, body mass index; WHtR, waist-to-height ratio; WHR, waist-to-hip ratio.

### 3.3 ROC analysis

**Figure 1:**
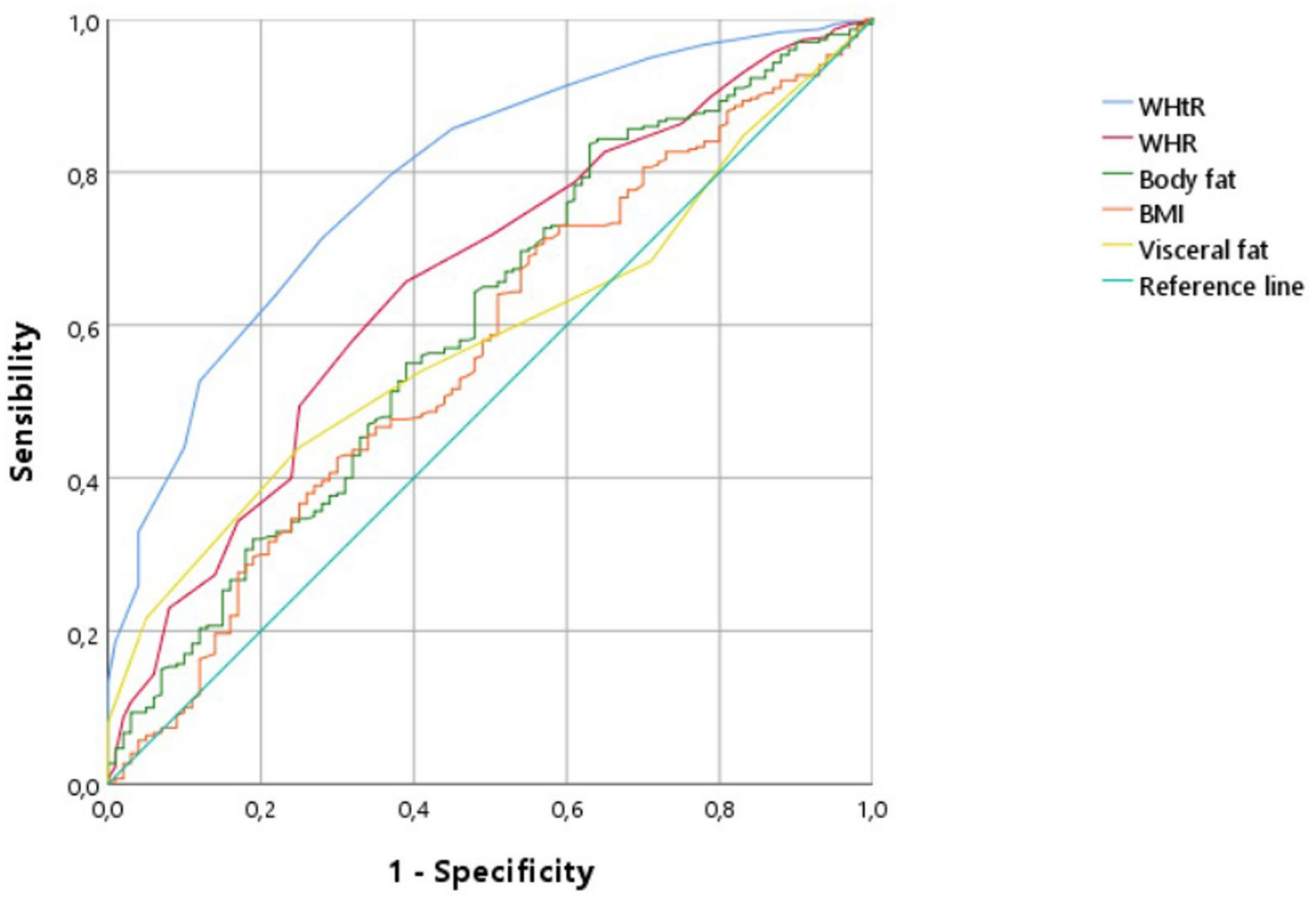
ROC curves comparing AUCs for all anthropometric indicators.

Receiver operating characteristic (ROC) curve analyses were conducted to evaluate and compare the discriminatory performance of anthropometric indices for identifying hypertension (Table 4). WHtR demonstrated the highest diagnostic accuracy, with an area under the curve (AUC) of 0.793 (95% CI: 0.744–0.842), indicating good discriminatory capacity. In contrast, WHR showed moderate performance (AUC = 0.657; 95% CI: 0.596–0.718), while body fat percentage (AUC = 0.603; 95% CI: 0.538–0.668), visceral fat level (AUC = 0.584; 95% CI: 0.525–0.642), and BMI (AUC = 0.568; 95% CI: 0.503–0.633) demonstrated comparatively lower discriminatory ability (Figure 1).

**Table 4:** ROC curve analysis of anthropometric indicators for predicting hypertension.

Indicator	AUC (%CI)	*p*	Cut-off point	Sensitivity (%)	Specificity (%)	Youden’s index
BMI (kg/m^2^)	0.568 (0.503–0.633)	0.042	24.09	79.3	30.0	0.093
WHtR	0.793 (0.744–0.842)	0.000	0.505	79.7	63.0	0.427
WHR	0.657 (0.596–0.718)	0.000	0.805	86.3	25.0	0.113
Body fat (%)	0.603 (0.538–0.668)	0.002	26.12	73.7	40.0	0.137
Visceral fat level	0.584 (0.525–0.642)	0.012	9.5	21.7	95.0	0.167

## 4 Discussion

This study aimed to determine the prevalence of overweight and obesity using both general and central anthropometric indicators and to identify the most reliable anthropometric measure for predicting hypertension among Moroccan adults. The principal finding is that WHtR presented the strongest independent association with hypertension and the highest discriminatory performance. The distribution of BMI categories revealed a high prevalence of excess body weight in the study population, with 58.3% of participants classified as overweight and 8.8% as obese, corresponding to an overall prevalence of 67.1% for excess body weight. Importantly, individuals with hypertension exhibited a mean BMI of 26.3 ± 2.8 kg/m^2^, which falls within the overweight range. WHtR demonstrated the strongest independent association with hypertension (ORa = 6.79, 95% CI: 3.57–12.90, *p* < 0.001). Furthermore, ROC analysis confirmed its superior discriminatory performance (AUC = 0.793), outperforming BMI and other adiposity indicators. These results provide important evidence from a Moroccan population and align with an expanding body of international literature demonstrating the superiority of WHtR over BMI in predicting hypertension risk (29, 30). Participants with hypertension were significantly older than normotensive individuals, consistent with age-related increases in arterial stiffness, endothelial dysfunction, and cumulative cardiometabolic burden (30–32). Although sex differences in hypertension have been reported elsewhere (15, 33), no significant association was observed in our cohort. Differences in education and marital status likely reflect broader socioeconomic gradients influencing cardiovascular risk through disparities in health literacy, access to care, and exposure to behavioral risk factors (34).

These sociodemographic variations underscore the importance of accounting for potential confounders. Accordingly, multivariable models were adjusted for age, sex, education, income, smoking, alcohol consumption, and physical activity. The persistence of the WHtR-hypertension association after adjustment supports its independent contribution to blood pressure dysregulation.

The observed superiority of WHtR as a predictor of hypertension may be attributed to its capacity to more accurately represent abdominal fat accumulation relative to overall body size. Visceral adiposity is metabolically active and contributes to insulin resistance, chronic low-grade inflammation, and adipokine imbalance (35). It promotes activation of the sympathetic nervous system and the renin-angiotensin-aldosterone system, leading to sodium retention, vascular remodeling, and increased peripheral resistance (15, 35, 36). Central obesity is also associated with endothelial dysfunction, oxidative stress, and arterial stiffness key mechanisms in the development and maintenance of hypertension (14, 37).

By normalizing waist circumference to height, WHtR more accurately reflects abdominal fat distribution relative to body size and reduces the misclassification frequently observed with BMI (38). In African and North African contexts undergoing rapid epidemiological transition (39), central adiposity is increasingly prevalent even among individuals with normal BMI (40, 41).

Our findings are consistent with studies conducted in Asian, European, and African populations reporting higher AUC values for WHtR compared with BMI and WHR (30, 42, 43). The optimal cut-off identified in this study (0.505) closely approximates the widely reported threshold of 0.5 across diverse ethnic groups (44). However, data from North African populations remain limited. Evidence from Moroccan populations also highlights the growing burden of hypertension and its relationship with adiposity indicators. A large retrospective analysis conducted in Rabat reported a high prevalence and severity of hypertension, particularly among older adults, emphasizing the magnitude of this public health challenge in Morocco (45). In addition, a cross-sectional study conducted in Khouribga demonstrated that abdominal obesity assessed by waist-to-hip ratio was significantly associated with hypertension (46). These findings are consistent with the present study and reinforce the importance of central adiposity indicators for identifying individuals at increased cardiovascular risk in Moroccan populations.

From a public health perspective, these findings have important implications for hypertension prevention and early detection strategies in Morocco. Given its simplicity, low cost, and ease of measurement, the WHtR could be incorporated into routine screening practices within primary healthcare services and community-based prevention programs. Integrating WHtR assessment into community-based screening initiatives conducted in primary healthcare centers could facilitate early identification of individuals at elevated cardiovascular risk. Such strategies could complement existing national non-communicable disease prevention programs by enabling targeted lifestyle interventions, including physical activity promotion, weight management, and dietary counseling among individuals with elevated WHtR values. By promoting early risk detection and preventive action, the incorporation of WHtR into public health practice may contribute to improved hypertension control and reduction of cardiovascular disease burden in Morocco.

Several limitations should be acknowledged when interpreting these findings. First, the cross-sectional design does not allow assessment of temporal relationships between anthropometric indicators and hypertension. Second, participants were recruited from a single province, which may limit the generalizability of the results to other Moroccan or North African populations. Third, body composition was assessed using bioelectrical impedance analysis, which provides indirect estimates of adiposity and visceral fat and may be influenced by hydration status and physiological variability. Despite these limitations, to the best of our knowledge, this is the first study conducted in Morocco to comprehensively and simultaneously evaluate a wide range of anthropometric and body composition indicators in relation to hypertension. The use of multivariable models adjusted for an extensive set of sociodemographic and lifestyle factors strengthens the internal validity of the findings. Moreover, the combined application of logistic regression and ROC analyses provides complementary insights into both etiological relevance and practical screening utility. Importantly, the identification of a population-specific WHtR cut-off further enhances the public-health relevance of these results. Future research should validate these findings using longitudinal designs to assess the predictive value of WHtR for incident hypertension. Additionally, integrating simple anthropometric indicators with biochemical or genetic markers may further improve hypertension risk stratification.

The finding in this cross-sectional study suggest that central adiposity, as assessed by the WHtR is a clinically relevant predictor of hypertension than conventional measures of general obesity. After adjustment for key sociodemographic and lifestyle factors, WHtR demonstrated the strongest independent association with hypertension and showed superior discriminatory performance compared with other anthropometric indicators. The optimal WHtR cut-off value of 0.5 provided a favorable balance between sensitivity and specificity, highlighting its potential usefulness for both clinical screening and population-based prevention strategies. Given its simplicity, low cost, and ease of measurement, WHtR can be readily incorporated into routine health assessments to improve early identification of individuals at high risk of hypertension. These findings emphasize the importance of considering fat distribution rather than overall adiposity in cardiovascular risk assessment. However, longitudinal prospective studies are needed to confirm the predictive value of WHtR for incident hypertension and cardiovascular outcomes and to determine whether these associations remain consistent across different demographic and clinical subgroups.

## Data Availability

The raw data supporting the conclusions of this article will be made available by the authors, without undue reservation.
